# Evaluation of methods to concentrate and purify ocean virus communities through comparative, replicated metagenomics

**DOI:** 10.1111/j.1462-2920.2012.02836.x

**Published:** 2013-05

**Authors:** Bonnie L Hurwitz, Li Deng, Bonnie T Poulos, Matthew B Sullivan

**Affiliations:** Ecology and Evolutionary Biology, University of ArizonaTucson, AZ 85721, USA

## Abstract

Viruses have global impact through mortality, nutrient cycling and horizontal gene transfer, yet their study is limited by complex methodologies with little validation. Here, we use triplicate metagenomes to compare common aquatic viral concentration and purification methods across four combinations as follows: (i) tangential flow filtration (TFF) and DNase + CsCl, (ii) FeCl_3_ precipitation and DNase, (iii) FeCl_3_ precipitation and DNase + CsCl and (iv) FeCl_3_ precipitation and DNase + sucrose. Taxonomic data (30% of reads) suggested that purification methods were statistically indistinguishable at any taxonomic level while concentration methods were significantly different at family and genus levels. Specifically, TFF-concentrated viral metagenomes had significantly fewer abundant viral types (*Podoviridae* and *Phycodnaviridae*) and more variability among *Myoviridae* than FeCl_3_-precipitated viral metagenomes. More comprehensive analyses using protein clusters (66% of reads) and k-mers (100% of reads) showed 50–53% of these data were common to all four methods, and revealed trace bacterial DNA contamination in TFF-concentrated metagenomes and one of three replicates concentrated using FeCl_3_ and purified by DNase alone. Shared k-mer analyses also revealed that polymerases used in amplification impact the resulting metagenomes, with TaKaRa enriching for ‘rare’ reads relative to PfuTurbo. Together these results provide empirical data for making experimental design decisions in culture-independent viral ecology studies.

## Introduction

Viruses are the most abundant and diverse biological entities on the planet (Wommack and Colwell, 2000; Rohwer, 2003). Their impact is global: affecting microbial hosts through mortality, remineralization of nutrients and horizontal gene transfer (reviewed in Fuhrman, 1999; 2000; Wommack and Colwell, 2000; Weinbauer, 2004; Suttle, 2005; 2007; Breitbart *et al*., 2007). They can even drive the evolutionary trajectory of the Earth's fundamental biogeochemical processes by encoding ‘host’ genes (Mann *et al*., 2003; Lindell *et al*., 2004; Sullivan *et al*., 2005; 2006; Sharon *et al*., 2007) that are expressed during infection (Lindell *et al*., 2005; Clokie *et al*., 2006; Dammeyer *et al*., 2008) and confer a direct fitness advantage for the phage (Bragg and Chisholm, 2008; Hellweger, 2009). Such viral-encoded photosynthesis genes likely also have significant ecological and evolutionary impact on ocean ecosystems as they dominate global ocean microbial metagenomes (Sharon *et al*., 2007) and alter the long-term evolutionary trajectories of both phage and host copies of the gene (Sullivan *et al*., 2006).

Despite the abundance of viruses in aquatic marine environments, studying viruses in the wild is fraught with methodological challenges. Perhaps the most notable is that wild viruses and their microbial hosts are rarely cultivable (Edwards and Rohwer, 2005) and the field is reliant upon culture-independent methods such as metagenomics wherein the majority of reads (50–90%) show no significant similarity to a sequence within a known organism (Breitbart *et al*., 2002; Angly *et al*., 2006; Bench *et al*., 2007; Dinsdale *et al*., 2008; Williamson *et al*., 2008). Furthermore, to create metagenomes, viral particles must be concentrated and purified from small volumes of filtrate while minimizing contamination and artefact (Lawrence and Steward, 2010; Wommack *et al*., 2010). We previously introduced a new method, FeCl_3_ precipitation, for concentrating viruses from seawater (John *et al*., 2011) that was more efficient than the standard method of tangential flow filtration (TFF).

While little is known about the effects of sample processing and library construction procedures on viral metagenomes several studies have addressed experimental issues in microbial metagenomics. First, variable DNA extraction efficiencies across microbes in the environment are likely due to variation in cell wall and membrane structure (Carrigg *et al*., 2007). Second, amplification protocols used to bolster limiting quantities of DNA, such as multiple displacement amplification (MDA), are not quantitative due to amplification bias (Yilmaz *et al*., 2010). Third, fosmid cloning can also bias the representation of species (Temperton *et al*., 2009). Finally, *in silico* processing is problematic where organisms are more divergent than those in databases (McHardy and Rigoutsos, 2007). Summarily, even in the case of *in vitro* metagenomic simulations where both the organisms and abundances were known *a priori*, sequence coverage variation was observed, and ascribed to differences in ‘growth conditions, organismal growth phase, DNA extraction efficiency, cloning bias, sequencing efficiency, or relative genome copy number’ (Morgan *et al*., 2010). Thus rigorous, systematic, empirical studies are needed to take us one step closer to being able to meaningfully cross-compare metagenomic data sets generated using different methods.

Here, we evaluate the effect of commonly used aquatic viral concentration and purification protocols on triplicate metagenomes across four different method combinations. Specifically, seawater collected from the site of the first viral metagenomes, San Diego's Scripps Pier (Breitbart *et al*., 2002), was used to generate triplicate viral metagenomes to evaluate taxonomic and protein cluster variability across two concentration (TFF and FeCl_3_ precipitation) and three purification methods (DNase only, DNase + CsCl and DNase + sucrose) in four method combinations as follows: (i) TFF and DNase + CsCl, (ii) FeCl_3_ precipitation and DNase only, (iii) FeCl_3_ precipitation and DNase + CsCl and (iv) FeCl_3_ precipitation and DNase + sucrose.

## Results

**Fig. 1:**
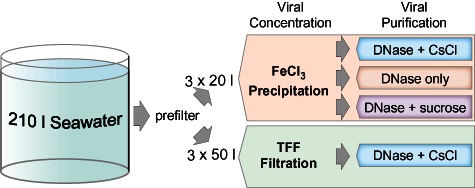
Workflow showing the process of creating each viral metagenomic replicate using different concentration (TFF or FeCl_3_) and purification methods (DNase only, DNase + CsCl or DNase + sucrose) from a 210 l sample of seawater taken from Scripps Pier, San Diego, CA. The ‘3 ×’ refers to that each subsample was processed independently from the 210 l pooled initial sample.

Thirteen metagenomes were generated for this study from Scripps Pier, San Diego, California Pacific Ocean seawater: a microbial metagenome from the 0.2–2.7 µm size fraction, and 12 viral metagenomes derived from the < 0.2 µm size fraction. The viral metagenomes were produced using four standard protocols for viral concentration and purification (TFF DNase + CsCl, FeCl_3_ DNase only, FeCl_3_ DNase + CsCl and FeCl_3_ DNase + sucrose) in triplicate from the same seawater sample (Fig. 1). Triplicate metagenomes were used to rigorously evaluate *intra*- versus *inter*-method variability and document any taxon-specific or protein diversity biases associated with these methods. The replicated metagenomes are abbreviated as TC1, TC2, TC3 (TC; TFF DNase + CsCl), FD1, FD2, FD3 (FD; FeCl_3_ DNase only), FC1, FC2, FC3 (FC; FeCl_3_ DNase + CsCl) and FS1, FS2, FS3 (FS; FeCl_3_ DNase + sucrose) for simplicity.

### Community composition and variability across methods

A one-way analysis of variance was conducted to evaluate the relationship between viral concentration and purification methods based on the hit count at the superkingdom, family and genus levels (see *Experimental procedures*, Table S1). At the broadest taxonomic level (superkingdom), we observed no significant differences across concentration or purification methods other than slightly more variability between replicate metagenomes for TC and FS (Fig. S1, Table S1). Overall, 66–81% of the reads in viral metagenomes and 37% in the microbial metagenome had no significant hit to anything in available databases (see *Experimental procedures*). Of the subset of reads from viral metagenomes with significant hits, 44–63% had no superkingdom designation in the database, little to no reads were classified as ‘Archaea’, 11–14% were classified as ‘Bacteria’, 2–3% as ‘Eukaryota’ and 22–42% as ‘Viruses’. Notably, these percentages include a small correction (∼ 1%) to reclassify abundant ‘prophage’ and ‘AMG’ (auxiliary metabolic gene) sequences that get misclassified as ‘bacterial’ (see *Experimental procedures*). As expected, these superkingdom-level taxonomic findings contrasted those from the microbial metagenome where the bulk of the identified reads were ‘Bacteria’ (47%) and many fewer were ‘Viruses’ (3%), suggesting that the metagenomes from viral-size-fractionated seawater were indeed enriched for viruses regardless of concentration or purification method.

**Fig. 2:**
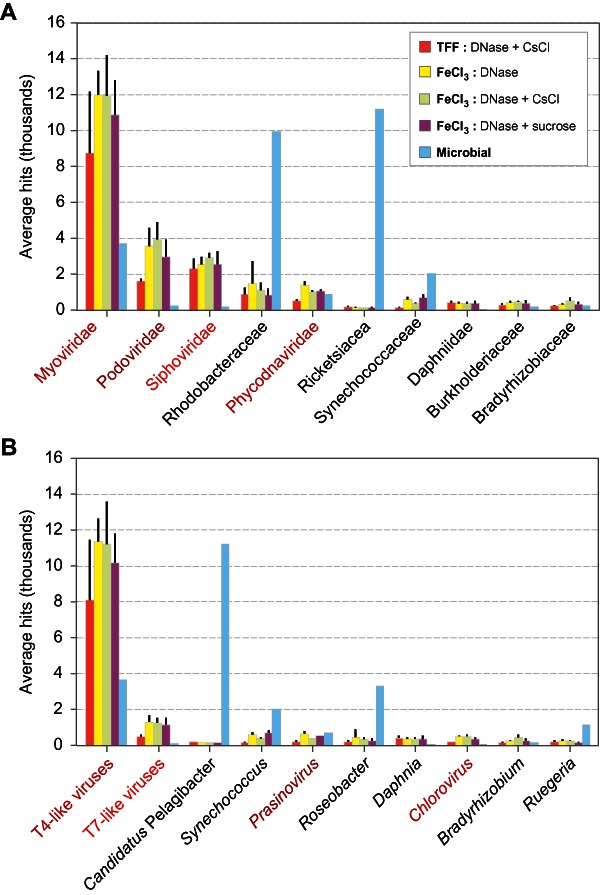
Rank abundance curve of (A) family-level (B) and genus-level taxonomy for the top 10 taxa observed in the data. Only four family-level and four genus-level taxa are viral (highlighted with red text); the remaining are microbial taxa.

With increasing taxonomic resolution at the family and genus levels respectively (Fig. 2), we found relatively consistent rank abundance profiles across all methods for the top 10 taxonomic designations. These top 10 taxonomic distributions represent 76–87% of the total reads that had a taxonomic designation at the family level and 52–72% at the genus level, thus capturing a sizable fraction of our annotated data set. Increasing the taxa to the top 30 only negligibly added more reads. We found significant differences in the absolute count for the more abundant viral taxa at the family and genus levels (Table S1). With respect to the concentration method, metagenomes derived from TC viruses significantly underrepresented the *Podo*- and *Phycodna*- (Prasino- and Chloroviruses) *viridae* relative to FD, FC and FS viral concentrates. Also, the TC samples showed more variability in the absolute counts for the most abundant viral type, *Myoviridae* (T4-like viruses), as compared with FD, FC and FS (Table S1). The choice of purification method (FD, FC and FS), however, did not have a significant effect on the distribution of hits at any taxonomic level. The most notable difference was that *Rhodobacteriaceae* was morevariable in the FD replicates as compared with FC and FS viral concentrates.

### Diversity of protein clusters

Given limited available annotation, we next explored a greater fraction of our data using protein clusters to estimate sequencing effort and protein diversity across methods (see *Experimental procedures*). When we clustered the ∼ 1.8 million total open reading frames (ORFs) from all of the viral methods, we found that 27% clustered with existing protein clusters from the Global Ocean Survey (GOS), 12% clustered in novel protein clusters with 20 + members, 40% with 2–19 members and 21% could not be placed in protein clusters at all. Our final set of 20 + high-confidence protein clusters consisted of 6845 GOS protein clusters and 6178 novel protein clusters. When we mapped our reads back to the ORFs in high-confidence protein clusters 66% of reads matched.

To test the ‘replicability’ of each method, we examined the ORF membership of each of the 20 + protein clusters with the null hypothesis that each method would be represented by at least one ORF in these abundant protein clusters. Overall, we found that 53% of clusters contained ORFs from all four methods (Table 1). Of the remaining clusters, 13% were found in TFF-concentrated samples (TC) and 11% in FeCl_3_-precipitated samples (FD, FC and FS) only and may represent clusters specific to the concentration methods. In total, 82% of clusters contained ORFs from at least two methods indicating that most clusters were not specific to a single method and are likely to represent real viral proteins in the sample rather than artefact.

**Table 1:** A compositional analysis of ORFs in 20 + protein clusters by concentration and purification method

Methods represented	# clusters	% clusters
FC&FD&FS&TC	6861	52.7
TC	1672	12.8
FC&FD&FS	1364	10.5
FD	761	5.8
FD&TC	492	3.8
FC&FD&TC	394	3.0
FC	271	2.1
FD&FS&TC	221	1.7
FS	203	1.6
FC&TC	187	1.4
FC&FD	171	1.3
FD&FS	161	1.2
FS&TC	120	0.9
FC&FS&TC	114	0.9
FC&FS	31	0.2

The following abbreviations are used for each method: FC = FeCl_3_ CsCl + DNase, FS = FeCl_3_ sucrose + DNase, FD = FeCl_3_ DNase, TC = TFF and CsCl + DNase and TF = TFF CsCl + DNase.

**Fig. 3:**
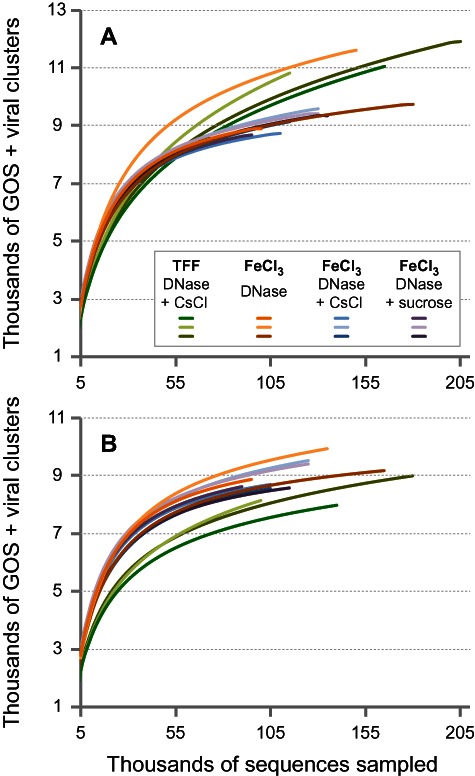
Rarefaction analysis of hits to protein clusters from each viral metagenome using (A) all sequences and (B) abundant (k-mer > 1) sequences. To be conservative, only protein clusters with > 20 members were used in these analyses.

We then explored the data from the high-confidence protein clusters (GOS clusters + novel clusters with 20 + members) using a rarefaction analysis to examine protein diversity in each sample and replicate. These analyses suggested that our sampling was relatively deep, but that four samples (TC1, TC2, TC3 and FD2) had greater protein diversity than other methods (Fig. 3A).

### Shared k-mer analysis of reads to distinguish methods and replicates

**Fig. 4:**
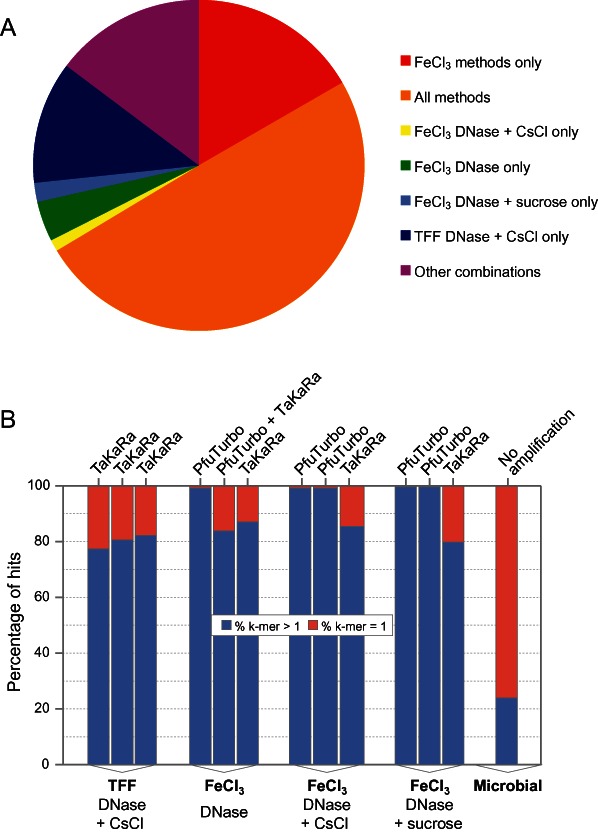
K-mer-based analysis of shared reads between methods and replicates. (A) Percentage of total reads that are shared between methods based on a k-mer-based analysis and (B) percentage of ‘rare’ (k-mer = 1) versus abundant sequences (k-mer > 1) in each of the four viral metagenomic methods and a non-replicated microbial metagenome. The enzymes used for linker amplification (TaKaRa or PfuTurbo) are listed above each sample. The microbial sample includes more ‘rare’ sequences because the diversity in the sample is undersampled based upon rarefaction analysis (data not shown).

In order to better quantify and separate out distinct reads in each method or replicate that drive differences in the rarefaction curves (above), we compared and contrasted k-mers in reads for all samples representing 100% of our data set (Fig. 4A; see *Experimental procedures*). Overall, the k-mer analysis mirrored the results from the protein clustering analysis and showed that 50% of reads are shared between all methods and 82% are shared with at least one other method. Also comparably, 12% were found in just TC and 17% in FeCl_3_-precipitated samples (FD, FC and FS), indicating that each subset of reads may be specific to each concentration protocol as noted previously.

To explore what drives the differences in the rarefaction curves we examined the fraction of ‘rare’ sequences (k-mer = 1; Fig. 4B) in the metagenomes, and found considerable variation both within and across methods. This variability appears to be driven by the enzyme used in linker amplification, as samples using the TaKaRa polymerase had a higher percentage of rare sequences (> 13%) than those using PfuTurbo (< 1%). This was especially apparent in FD2, which was prepared twice for sequencing, once with PfuTurbo and a second time with TaKaRa and then combined into one sample. Here, the fraction of rare sequences in the metagenomes was 0.4% and 22% for PfuTurbo and TaKaRa respectively. When the ‘rare’ sequences were removed from all samples in the rarefaction analysis, the TFF replicates showed the least diversity (Fig. 3B).

**Fig. 5:**
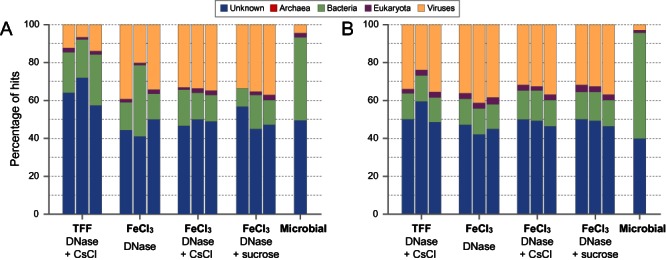
Superkingdom taxonomic profile of reads for each triplicate sample from the four viral metagenome methods and a non-replicated microbial metagenome for (A) rare (k-mer = 1) and (B) abundant (k-mer > 1) reads.

The increased percentage of rare sequences, however, does not fully explain the difference in the rarefaction curves, as other replicates were also processed using the TaKaRa enzyme and had a similarly high fraction of rare sequences. To further explain the differences, we taxonomically classified (by superkingdom) the metagenome reads that were unique and shared in each method (Fig. 5). We found that ‘rare’ sequences (k-mer = 1) in these anomalously diverse samples were enriched for ‘bacteria’ (15–22% in TC1, TC2, TC3, FD2 versus 5–9% for all other samples) and suppressed for ‘virus’ (5–11% in these four samples versus 9–20% for all other samples; Fig. 5A). In contrast, the category rankings among ‘non-rare’ (k-mer > 1) sequences did not vary across methods (Fig. 5B). We also found that the ratio of annotated viral to bacterial reads is less than one in the subset of reads specific to each replicate suspected of bacterial contamination (TC1, TC2, TC3, FD2) and much greater than this (commonly > 2) for the other treatments or replicates (Table 2).

**Table 2:** Reads that are exclusive to a certain method and replicate based on a k-mer analysis and their taxonomic assignment to bacteria and viruses

Sample	Replicate	Enzyme	Total reads	Exclusive reads	% exclusive reads	Exclusive reads bacteria	Exclusive reads viruses	Ratio viruses/bacteria
FeCl_3_ DNase + CsCl only	1	PFU-Turbo	141 000	1 004	0.7	16	36	2.3
FeCl_3_ DNase + CsCl only	2	PFU-Turbo	175 119	861	0.5	24	52	2.2
FeCl_3_ DNase + CsCl only	3	TaKaRa	171 220	26 011	15.2	855	1987	2.3
FeCl_3_ DNase only	1	PFU-Turbo	134 504	1 697	1.3	36	95	2.6
FeCl_3_ DNase only	2	PFU-Turbo + TaKaRa	236 591	45 075	19.1	3707	2571	0.7
FeCl_3_ DNase only	3	TaKaRa	274 368	49 542	18.1	1356	4063	3.0
FeCl_3_ DNase + sucrose only	1	PFU-Turbo	122 151	437	0.4	5	13	2.6
FeCl_3_ DNase + sucrose only	2	PFU-Turbo	158 816	492	0.3	13	18	1.4
FeCl_3_ DNase + sucrose only	3	TaKaRa	223 859	45 105	20.1	829	2020	2.4
TFF DNase + CsCl only	1	TaKaRa	308 510	120 430	39.0	4570	3954	0.9
TFF DNase + CsCl only	2	TaKaRa	193 113	71 512	37.0	4367	1885	0.4
TFF DNase + CsCl only	3	TaKaRa	319 781	102 022	31.9	6669	5713	0.9

Further, when we mapped the metagenome reads to five abundant microbial genomes, we found that the three anomalously diverse TFF replicates (TC1, TC2 and TC3) contained more recruitment to and spread throughout three of the five microbial genomes (alphaproteobacterium BAL199, and gammaproteobacteria HTCC 2143 and HTCC 2148) (Fig. S2). This pattern contrasted that observed for the other methods where little to no recruitment was observed except in the microbial metagenome where the level and spread of recruitment was similar (Fig. S2). Reads that mapped to the two other abundant microbial genomes displayed a different pattern: (i) *alphaproteobacterium HIMB114* hits clustered in ∼ 50 kb region for all viral metagenomes across all methods, but not for the microbial metagenome while (ii) hits to *Daphnia pulex* were present in just two genes across all viral and microbial metagenomes (Fig. S2). We interpret these latter findings to represent prophages and viral-encoded AMGs (*sensu*; Breitbart *et al*., 2007), while the former findings suggest that ‘rare’ sequences in TC1, TC2 and TC3 represented trace microbial DNA contamination that inflated their rarefaction curves. Despite having an increased percentage of bacterial hits relative to the other replicates, FD2 did not show the same pattern of recruitment as TC1, TC2 and TC3 to the abundant microbes. Notably, however, FD2 had three to fourfold more hits to *Rhodobacteriaceae* as compared with FD1 and FD3 (Fig. S2), and it is likely to be sporadically contaminated by bacteria (potentially during amplification) from this family or other less abundant bacteria not analysed here.

Because some replicates had as many as 23% rare sequences, we sought to determine whether these sequences were ‘real’ or artefact. To do this, we examined two key features of their hits to protein clusters: percent identity and coverage. While rare sequences hit protein clusters with a lower-percentage identity than their more abundant counterparts, the coverage of the hits was consistent except in the case of FD2 where sequences with 100% coverage increased from 16% in abundant sequences to 27% among rare sequences. Although the rare sequences map at lower-percentage identity, we interpret this to be due to the fact that ‘rare’ organisms are not well represented in our protein clusters (required > 20 members for *bona fide* clusters) or any database (most studies sample only the dominant organisms). Given these results, we posit that these rare sequences are indeed ‘real’.

## Discussion

Here we find that the taxonomic signal inferred from the annotatable portion of viral metagenomes is relatively robust to variations in commonly used sample concentration and purification procedures (Lawrence and Steward, 2010; Wommack *et al*., 2010) with deviations now well documented. When looking at a larger fraction of our data set using protein clustering (66% of reads) and k-mers (100% of reads), we find that our sampling effort is relatively deep and the majority of protein clusters and reads are shared with at least one other method (82% and 81%) and (50% and 53%) are shared among all. Thus, small-scale differences define these methods.

To this end, we caution researchers on three fronts. First, if desiring bacteria-free viral metagenomes, then avoidance of TFF concentration and DNase-only purification methods may help. Even though all methods were relatively rigorous in extracting viruses, we observed microbial DNA contamination in trace amounts predominantly in the ‘rare’ (k-mer = 1) component of TFF-concentrated samples, as well as one replicate of the DNase-only purification samples. Second, if interested in studying particular viral taxa, care should be taken in choosing concentration and purification methods as some taxa, while not undersampled to the point of altering rank abundance, may be underrepresented by some methods. We note here that our data sets were not rich in the larger eukaryotic viruses (Yoshida *et al*., 2011) that may be due to undersampling in this study as a result of a 0.2 µm prefiltration step to create the ‘viral size fraction’. Further, it is possible that TFF may have allowed some smaller viruses (e.g. podoviruses and non-tailed viruses) to pass through the 100 kDa filter. Empirical data from Pacific Ocean viral communities suggest that < 0.3% of the total viruses pass through these filters (J. Brum, pers. comm.). If all of viruses that passed through filtration were *podoviruses* this could explain the reduction in *podoviruses* we found in the TFF samples as compared with other samples that also represented ∼ 0.3% of reads in the TFF samples. Overall, viral loss in permeate should only minimally impact the taxonomic assessments described here, given that 100 kDa filter pore sizes are only ∼ 10 nm and the smallest known ocean viruses are 20 nm. Tangential flow filtration concentration set-ups of 30 kDa and 50 kDa used in previous marine viral ecology studies may have further reduced loss of small viruses.

Third, it is important to consider amplification options when preparing environmental viral metagenomes. The metagenomes in this study were generated from DNA that was linker-amplified using protocols optimized for quantitative metagenomics from next-generation sequencing (see companion paper Duhaime *et al*., 2012). We document here that the choice of polymerase greatly impacts your access to ‘rares’ in the community, but in a systematic manner. Mechanistically, we speculate that late cycle PCR dCTP deamination to dUTP inhibits amplification of dominant templates in the TaKaRa reactions thus selecting for rare templates not yet deaminated. Such issues are not encountered in the PfuTurbo reactions because it contains an enzyme to convert dUTP products to dUMP that allows dominant templates to be processed in proportions relative to their actual occurrence in the population. Further, while linker amplification methods have a slight *systematic* (%G+C) bias (Duhaime *et al*., 2012), they are incredibly precise as evidenced by minimal variation between replicates which allows for quantitative cross-comparison between samples. In contrast, other published viral metagenomic data sets suffer from methodological issues not recognized at the time of publication – either being small and biased by cloning (e.g. linker-amplified and Sanger-sequenced; Breitbart *et al*., 2002; 2003; 2004; Bench *et al*., 2007) or resulting from whole genome-amplified DNA (Angly *et al*., 2006; Dinsdale *et al*., 2008) which is now known to have unpredictable, *stochastic* biases that lead to non-quantitative metagenomic data sets (Yilmaz *et al*., 2010), as well as *systematic* biases of particular relevance to viruses (Kim *et al*., 2008; Kim and Bae, 2011).

Finally, the ‘bacterial’ signal in viral metagenomes presents an area where informatics solutions are greatly needed. Specifically, viral metagenomes commonly contain up to ∼ 1/3 ‘bacterial’ sequences (e.g. Breitbart *et al*., 2002; 2003; Angly *et al*., 2006; Bench *et al*., 2007; Cantalupo *et al*., 2011) which are loosely attributed to ‘auxiliary metabolic genes’ or host genes legitimately in viruses (*sensu*; Breitbart *et al*., 2007), prophages in microbial genomes that are yet to be annotated or microbial DNA that is mispackaged in viral capsids in elements called gene transfer agents (GTAs; Lang and Beatty, 2007; Stanton, 2007; Biers *et al*., 2008). Here we present a new means to quantify the relative proportion of ‘bacterial’ hits that are prophage through examining the distribution of reads mapping to abundant bacterial reference genomes; we find that prophages contribute a relatively small fraction (only 1%) which is in line with the fraction of microbial genomes devoted to identifiable prophages (e.g. Casjens, 2003). While columns and reagents can be contaminated with low levels of bacteria and mouse sequences (van der Zee *et al*., 2002; Evans *et al*., 2003; Shen *et al*., 2006; Erlwein *et al*., 2011), non-uniform contamination across treatments and replicates argues against kit-based contamination being responsible. Further, it is unlikely that contaminating DNA would have survived myriad purification methods across triplicate samples, which suggests that GTAs are the probable largest contributor to the ‘bacterial’ signal in these data.

### Conclusions

The data and analyses presented here establish a quantitative framework for researchers to more rigorously understand and plan for biases in the sequence-based methods used to compare viral communities over space and time. Notably, however, our work is limited to dsDNA viruses and there likely remain many biases to be rigorously investigated in viral ecology. For example, new copurification methods allow simultaneous access to RNA and DNA viruses from the same sample (Andrews-Pfannkoch *et al*., 2010). While this is a giant step forward for sampling, it remains an open question whether the method effectively purifies RNA and DNA viruses in a manner that preserves the relative representation of these viruses from the wild. Further, myriad sequencing and library preparation options now exist for generating viral metagenomes that begs the question of their intercomparability. With advancing sequencing and informatics technologies, quantitative evaluation becomes possible. Ultimately, as the field develops quantitative rigour with existing population level metrics, we will also migrate down the ‘single-entity genomics’ route (Allen *et al*., 2011) in the quest to map the population structure and quantify the relative abundance of viruses in wild communities. Our rigorous analysis of the current methodologies used for producing viral metagenomes complements these single-cell genomics efforts towards obtaining a less biased view of community composition and protein diversity.

## Experimental procedures

### Isolation of nucleic acid from SIO seawater microbial fraction

Approximately 200 l of seawater was filtered through a Whatman GF/D (2.7 µm) prefilter onto a Millipore Steripak GP20 (0.2 µm) filter unit after which 10 ml of 0.2 µm filtered sucrose lysis buffer (SLB, 50 mM TrisCl pH 8.0, 40 mM EDTA, 0.75 M sucrose) was added and the unit stored at −80°C until DNA extraction. Total nucleic acid was isolated using a modification of the protocol described in Frias-Lopez and colleagues (2008). Briefly, lysozyme (5 mg ml^−1^ in SLB) was added to the thawed unit to a final concentration of 0.5 mg ml^−1^. After incubation at 37°C for 30 min, 5 M NaCl (0.2 µm filtered) was added to a final concentration of 0.2 M. Proteinase K (20 mg ml^−1^) was then added (final concentration 0.5 mg ml^−1^) along with 10% SDS (0.2 µm filtered, final concentration 1%) and the unit incubated at 55°C for 20 min followed by 70°C for 5 min. The lysate was removed (∼ 14 ml) and extracted two times with phenol/chloroform (50:50 vol) equilibrated with TE followed by one extraction with chloroform. Phases were separated by centrifugation at 4°C for 5 min at 3320 *g* (4 K). Nucleic acid in the aqueous phase was concentrated using Amicon Ultra15 100 K MWCO filters (Millipore) to approximately 600 µl. Because the A_260/280_ ratio was below 1.8 when analysed by nanodrop, the nucleic acid was further purified with one phenol/chloroform (50:50 vol) extraction followed by one chloroform extraction. Thereafter, 3 M sodium acetate (0.2 µm filtered) was added (0.3 M final concentration) followed by 2.5 × volumes of 100% ethanol (0.2 µm filtered) to precipitate the nucleic acid overnight at −20°C. After centrifugation at 13 K for 20 min, the pellet was washed with 70% ethanol and air-dried. The pellet was resuspended in a total volume of 600 µl TE (10 mM TrisCl pH 8, 1 mM EDTA, 0.2 µm filtered). By nanodrop, the total microbial nucleic acid recovered was 1.6 µg with an OD_260/280_ of 1.99 and OD_260/230_ of 2.36.

### Collection, concentration and purification of viral community DNA

Approximately 210 l of surface seawater was collected from Scripps Pier (La Jolla, CA, USA; 7 April 2009) and prefiltered using a 150 mm GF/A filter (Whatman International, Maidstone, UK; Cat. #1820-150) and a 0.22 µm, 142 mm Express Plus filter (Millipore, Bellerica, MA, USA; Cat. #GPWP14250) with 20–50 l of filtrate haphazardly pooled into a 55 gallon trashcan that held ∼ 180 l at a time to minimize any potential variation between 20 l and 50 l carboys. The viruses in the filtrate were concentrated using either TFF or FeCl_3_ precipitation (FeCl_3_), the latter as in John and colleagues (2011). For the TFF method, triplicate 50 l subsamples were separately concentrated using a large-scale, 100 kDa TFF (Amersham Biosciences, Westborough, MA, USA; Cat. #UFP-100-C-9A) to 0.65–1 l followed by a small-scale, 100 kDa TFF (Millipore, Bellerica, MA, USA; Cat. #PXB100C50) to 12–14 ml; viruses were collected in the retentate after a final washing step. For the FeCl_3_ method, triplicate 20 l subsamples were subjected to a chemistry-based concentration method (John *et al*., 2011) where FeCl_3_ creates virus iron precipitates that canbe collected on 1.0 µm polycarbonate filters (GE Water and Process Technologies, Trevose, PA, USA; Cat. #K10CP14220) and resuspended in magnesium-EDTA-ascorbate buffer (0.1 M Mg_2_EDTA, 0.2 M ascorbic acid, pH 6.0) using 1 ml of buffer per 1 l of seawater. Resuspension was allowed to go overnight, rotating in the dark at 4°C, and the filters were transferred to fresh tubes and centrifuged for 5 min at low speed to collect the remaining fluid. The efficiency of recovery of viruses ranged from 18–26% using TFF to 92–95% using FeCl_3_ precipitation (John *et al*., 2011), as determined by SYBR Gold staining and epifluorescence microscopy (Noble and Fuhrman, 1998).

The resulting FeCl_3_ precipitation viral concentrates were purified using one of three methods – DNase only, DNase + CsCl or DNase + sucrose – with each 20 l split into three purification methods. In contrast, the TFF viral concentrates were only purified using DNase + CsCl. The ‘DNase-only’ method consisted of 100 U ml^−1^ DNase I (Roche, Indianapolis, IN, USA; Cat. #10-104-159-001) in reaction buffer (10 mM Tris-HCl pH 7.6, 2.5 mM MgCl_2_, 0.5 mM CaCl_2_) for 2 h at room temperature on a tube rotator; DNase I was inactivated by 100 mM (final concentration) of each EDTA and EGTA. The ‘DNase + CsCl’ method consisted of layering DNase I-treated viral concentrates on top of CsCl-step gradients [1.7, 1.56, 1.4, 1.2 g ml^−1^ in 100 kDa seawater permeate that had been autoclaved and 0.02 µm filtered, *sensu* (Thurber *et al*., 2009)], followed by centrifugation in a SW40ti rotor (Beckman) at 24 000 rpm (102 000 *g*) for 4 h, 10°C; viruses were harvested from fractions with densities of 1.4–1.52 g ml^−1^. Finally, the ‘DNase + sucrose’ method consisted of a 38% (w/v) sucrose ‘cushion’ prepared in 0.2 µm filtered SM buffer (50 mM Tris-HCl pH 7.5, 100 mM NaCl, 8 mM MgSO_4_) whereby DNase I-treated viral concentrate was layered on top at a ratio of one part sucrose to three parts viral concentrate. The tubes were centrifuged in a TH641 rotor (Sorvall) at 32 000 rpm (175 000 *g*) for 3 h, 18°C. The pellets beneath the sucrose cushion were collected in Tris-EDTA buffer (TE, 10 mM Tris-HCl pH 7.6, 1 mM EDTA) containing 100 mM each EDTA and EGTA (S.J. Williamson, pers. comm.).

### Extraction and linker amplification of viral community DNA

DNA was extracted from concentrated, purified viral particles using Wizard® PCR Preps DNA Purification Resin and Minicolumns (Promega, Madison, WI, USA; Cat. #A7181 and A7211 respectively) as previously described (Henn *et al*., 2010).

The DNA was prepared for sequencing using a linker amplification (LA) protocol modified from Henn and colleagues (2010). Briefly, DNA was sheared to a size of 400–800 bp using Covaris Adaptive Focused Acoustics (AFA) with the following conditions: 130 µl of DNA in TE buffer with up to 5 µg of total DNA, duty cycle of 5%, intensity of 3, 200 cycles per burst, for 62 s (E210). The DNA was concentrated to 35 µl using Microcon YM-100 centrifugal filter units (Millipore, Bellerica, MA, USA; Cat. #42412). The End-It DNA End-Repair kit (Epicentre Biotechnologies, Madison, WI, USA; Cat. #ER 81050) was used to end repair the sheared DNA. After clean-up using the Min-Elute Reaction Clean-up kit (Qiagen Sciences, Germantown, MD, USA; Cat. #28204), the DNA was ligated to a hemi-phosphorylated adaptor (Linker-A) using the Fast-Link DNA Ligation kit (Epicentre Biotechnologies, Madison, WI, USA; Cat. #LK 6201H). The double-stranded Linker A was prepared by annealing the single-stranded forward oligonucleotide (5′-phosphorylated-GTA TGC TTC GTG ATC TGT GTG GGT GT-3′) to the reverse oligonucleotide (5′-CCA CAC AGA TCA CGA AGC ATA C-3′). This was performed in TE (10 mM Tris-HCl pH 7.6, 1 mM EDTA) buffer supplemented with 50 mM NaCl. The DNA solution was heated in a water bath to 100°C for 5 min. The water bath was allowed to cool to room temperature and the annealed linker DNA was then placed on ice for 5 min. Linker A was diluted to 10 µM in nuclease-free water before use. After ligation, the DNA was immediately cleaned up using the MinElute Reaction Clean-up kit. Linker-ligated DNA was mixed with 6 × Blue/Orange Loading Dye (Promega, Madison, WI, USA; Cat. #G1881) and size-fractionated by gel electrophoresis in 1.5% SeaKem GTG agarose (Lonza, Rockland, MD, USA; Cat. #50071) prepared in sterile TAE (40 mM Tris-acetate, 2 mM EDTA) buffer and run at 80 V for 90 min. DNA markers placed in the outermost lanes were either Quick Load 100 bp DNA Ladder (New England Biolabs, Ipswich, MA, USA; Cat. #N0467S) or 1 kB-Plus DNA Ladder (Invitrogen, Carlsbad, CA, USA; Cat. #10787-026). The marker lanes were stained with ethidium bromide (1 ng µl^−1^) for 20 min and were used to as a guide to excise DNA in the range of 400–800 bp. DNA was extracted from the agarose slice using the Min-Elute Gel Extraction kit (Qiagen Sciences, Germantown, MD, USA; Cat. #28604).

The base sequence of the PCR phos-A primer used for amplification was 5′p-CCACACAGATCACGAAGCATAC-3′. Because multiple sources of DNA were to be pooled prior to library preparation for 454 pyrosequencing, 5 bp barcodes were introduced at the 5′ end of the primer so that the sequences from different sources could be identified from the data. The PfuTurbo Hotstart system (Stratagene, La Jolla, CA, USA; Cat. #600600) was used for amplification reactions. Reaction conditions were: 1–2 µl of Linker-A ligated, size-fractionated DNA, 12.5 µl of PfuTurbo Hotstart 2X Master Mix (0.1 U PfuTurbo per microlitre), 0.5 µl (5 pmol) of the 10 µM PCR phos-A primer, brought up to 25 µl with nuclease-free water (Promega, Madison, WI, USA; Cat. #P1193). Thermocycling conditions were denaturation at 95°C for 2 min, cycling for 25 to 30 cycles using 95°C for 30 s, 60°C for 60 s and 72°C for 90 s, and a final extension at 72°C for 10 min. Products were analysed on 1.5% agarose gels containing 0.5 ng µl^−1^ ethidium bromide, run in TAE buffer at 90 V for 30 min, using 5 µl of DNA. Amplified DNA was recovered from the PCR reaction mixes using the MinElute PCR Purification kit (Qiagen Sciences, Germantown, MD, USA; Cat. #28004) according to the manufacturer's directions. DNA was eluted off the mini-columns using 25–40 µl of the provided EB buffer warmed to 80°C. DNA was quantified using the Quant-iT Pico Green dsDNA assay kit (Invitrogen, Carlsbad, CA, USA; Cat. #P7589). Prior to sequencing library preparation, samples were pooled in equal amounts (generally considered molar equivalents due to shearing and size-fractionation steps).

### Sequencing viral community DNA

Metagenomic sequencing was performed using a GS-FLX Titanium system (454 Life Sciences, Roche, Mannheim, Germany) at the Broad Institute, Duke Institute for Genome Sciences and Policy, and the University of Arizona Genetics Core facility to generate ∼ 3.5 million reads for the 12 viral metagenomes and one microbial metagenome described above (Table 3). Reads were filtered to remove low-quality data based on protocols suggested by Huse and colleagues (2007). Briefly, reads were removed if they contained an ‘N’ anywhere in the sequence, were longer or shorter than two standard deviations from the mean sequence length or had a mean quality score less than two standard deviations from the mean quality score for all reads. Lastly, artificial duplicates from the pyrosequencing runs were removed using cd-hit-454 from the cd-hit package version 4.5.5 with default parameters (Niu *et al*., 2010). Based on these criteria, ∼ 2.6 million high-quality reads were retained for further analysis (Table 3). All sequences were deposited to CAMERA (http://camera.calit2.net) under the Project Accession Number CAM_P_0000914.

**Table 3:** Total sequences, QCed sequences and hits to SIMAP for each replicate in four viral metagenomic methods and a non-replicated microbial metagenome

Viral concentration method	Viral purification method	Replicate number	Total sequences	QCed sequences	Hits to SIMAP	% hits to SIMAP
FeCl_3_	DNase	1	172 745	134 504	41 582	30.9
FeCl_3_	DNase	2	345 858	236 591	60 428	25.5
FeCl_3_	DNase	3	428 258	274 368	79 238	28.9
FeCl_3_	DNase + CsCl	1	175 121	141 000	44 119	31.3
FeCl_3_	DNase + CsCl	2	218 300	175 119	59 322	33.9
FeCl_3_	DNase + CsCl	3	261 763	171 220	52 703	30.8
FeCl_3_	DNase + sucrose	1	162 078	122 151	39 724	32.5
FeCl_3_	DNase + sucrose	2	216 304	158 816	52 786	33.2
FeCl_3_	DNase + sucrose	3	308 139	223 859	43 436	19.4
TFF	DNase + CsCl	1	421 395	308 510	62 979	20.4
TFF	DNase + CsCl	2	264 916	193 113	60 408	31.3
TFF	DNase + CsCl	3	436 484	319 781	94 926	29.7
Microbial			136 227	100 704	63 252	62.8

### Taxonomic assignment of reads

Sequences that passed quality control were compared against the Similarity Matrix of Proteins (SIMAP) released on 25 June 2011 (Rattei *et al*., 2006) using BLASTX (Altschul *et al*., 1997). Hits were considered significant if they had an *E*-value of < 0.001. Although we did not impose a hit length cut-off, the majority of matches (99%) were between 100 and 500 bp in length with a mean length of 248 bp. To constrain our analyses to known organisms in SIMAP, the top 10 hits were analysed and sequences were assigned to the top hit that was not from an ‘uncultured’ organism. Taxonomic data at the species level were assigned based on the SIMAP hit, where the read inherited the taxonomy ID associated with the protein from SIMAP. Taxonomy data at the superfamily, family and genus levels were acquired using the NCBI taxonomy based on the species taxonomy ID. A subset of the NCBI taxonomy records for the most abundant viruses in the samples were manually curated to fill in missing data on the family and genus levels (http://www.eebweb.arizona.edu/faculty/mbsulli/scripts/hurwitz). We also updated NCBI taxa in our analyses for abundant bacteria that were missing annotation at the family level by assigning ‘*Candidatus Pelagibacter*’ to the family ‘*Ricketsiaceae*’ and ‘S*ynechococcus*’ to the family ‘S*ynechococcaceae*’.

### Prophage and AMG detection

To differentiate true bacterial hits from prophage regions in bacterial genomes or AMGs, we applied a secondary filter on sequences whose best match was bacterial in SIMAP. To do this, we constructed an in-house prophage database by comparing marine microbial genomes from the Gordon and Betty Moore Foundation to genes in viral genomes in NCBI and running Prophage Finder (http://bioinformatics.uwp.edu/∼phage/help.htm). The viral gene sequences were downloaded from NCBI on 7 July 2011, resulting in a total of 36 603 genes. The marine microbial genomes were downloaded from CAMERA (http://camera.calit2.net) on 7 July 2011 for a total of 19 459 genomic sequences. The microbial genomes were compared with the phage genes using BLASTX with an *E*-value cut-off of < 0.001 and a total of ∼ 1 million possible matches and alignments reported. Prophage regions were detected using Prophage Finder with default parameters. A total of 366 345 prophage protein sequences from 10 703 DNA fragments were found using this approach. To supplement these data, we also included data from Itai Sharon and colleagues on AMG sequences (Yooseph *et al*., 2007; Sharon *et al*., 2011).

### Statistical analyses of taxonomic distributions

Multiple statistical tests were conducted using a one-way analysis of variance (anova) to evaluate the relationship between viral concentration and purification methods and taxonomic groups. A separate test was performed for each of the top 10 taxonomic groups at the family and genus levels and all at the superkingdom level using Matlab (anova1) (Table S1). In each case, the independent variable was the method, and the dependent variable was the number of hits to the taxonomic group being tested. Because each of the methods and replicates had a variable number of total sequences, we normalized the hit count prior to our analysis by dividing the hits by the total sequences in the replicate and multiplying by 196 000 (the average number of sequences per library in 1000s). If the anova was deemed to be significant (*P*-value was < 0.05), we performed follow-up tests using the Tukey HSD test in Matlab (multcompare) to evaluate pairwise differences and identify means that differed between methods.

### Microbial genomic recruitment plots

To investigate whether hits to microbial genomes were from prophage or AMGs or sporadic microbial contamination, we created genomic recruitment plots for five abundant microbes: *alphaproteobacterium HIMB114*, *Daphnia pulex*, *alphaproteobacterium BAL199*, and *gammaproteobacteria HTCC 2143* and *HTCC 2148*. To do this, we compared sequences whose best BLAST match was to the aforementioned genomes to the contig sequences from each respective genome. Each hit was required to match with an *E*-value of < 0.001 and only the top match was retained. We used a Matlab script provided by Maureen Coleman to plot the blast data for the reads along a reference genome and calculate the coverage (http://www.eebweb.arizona.edu/faculty/mbsulli/scripts/hurwitz). Prophage regions were differentiated from AMGs because read coverage in these regions spanned > 4 kb in the microbial genome and was not confined to a single gene. Both AMG and prophage regions could be differentiated from sporadic contamination from microbial genomes based on a lack of alignment to the rest of the genome.

### K-mer analysis for discovering ‘rare’ sequences

Rare sequences (k-mer = 1) were distinguished from more abundant sequences (k-mer > 1) in the samples using vmatch version 2.1.5 (http://www.vmatch.de/). Specifically, we used mkvtree to create a suffix array for each sample, and then used vmerstat to search for the frequency of 20-mers in each of our metagenomic sequences with a minimum occurrence of 2 as compared with other sequences in the same sample. We parsed the vmatch data using a PERL script to assign a single frequency to each sequence based on the mode k-mer frequency of all of its 20-mer subsequences. The high-throughput data-processing pipeline containing the scripts for running these analyses is available here (http://www.eebweb.arizona.edu/faculty/mbsulli/scripts/hurwitz).

### Protein clustering and rarefaction

In order to find proteins, reads with a k-mer frequency > 1 were assembled into larger contigs using velvet version 1.0.15 (hash length = 29, -long) (Zerbino and Birney, 2008). Open reading frames were determined both on the individual reads (Table 3) and in assembled contigs using the metagenomic mode in Prodigal (Hyatt *et al*., 2010). Only non-redundant ORFs > 60 amino acids in length were retained. Protein sequences were clustered based on homology using cd-hit-v4.5.5-2011-03-31 (Niu *et al*., 2010) in a two-step process. First, we downloaded core cluster GOS (Yooseph *et al*., 2007) proteins from CAMERA (http://camera.calit2.net) and recruited sequences to known GOS protein clusters using cd-hit-2d (′-g 1 -n 4 -d 0 -T 24 -M 45000′). Sequences were considered to have a match if they hit with > 60% identity and > 80% coverage to the smallest sequence. Sequences that did not recruit to GOS protein clusters were then self-clustered using cd-hit with the same parameters as above. In total, our reads mapped to 11 116 GOS clusters and 6178 novel clusters with greater than 20 members. When we subtracted out data from the SIO microbial data set, our reads mapped to 6845 GOS clusters and 6178 novel clusters, with 449 980 and 210 451 reads respectively. The clustering pipeline containing the scripts for running these analyses is available here (http://www.eebweb.arizona.edu/faculty/mbsulli/scripts/hurwitz).

We compared all of the high-quality metagenomic reads in our data set (Table 3) with the sequences in the 20 + protein clusters using BLASTX (*E*-value < 0.001). Based on these data, we generated hit counts to the protein clusters and used the data for further rarefaction analysis using the rarefaction calculator (http://www.biology.ualberta.ca/jbrzusto/rarefact.php).

## Supporting information

**Fig. S1.** Superkingdom taxonomic profile across triplicate samples from each of the four viral metagenome methods and a non-replicated microbial metagenome. Note that these data represent only those metagenomic reads that had a significant hit to the SIMAP database.

**Fig. S2.** Fragment recruitment plots that show the distribution of metagenomic sequence reads that map to five abundant microbial genomes.

**Table S1.** The results of a one-way analysis ofvariance for the top taxonomic levels based on rank abundance inthe samples by superkingdom, family and genus. Significant resultsare shown in bold. Abbreviations are used for each method: FC =FeCl_3_ CsCl + DNase, FS = FeCl_3_ sucrose +DNase, FD = FeCl_3_ DNase, TC = TFF and CsCl + DNase and TF = TFF CsCl + DNase.

## References

[b1] Allen LZ, Ishoey T, Novotny MA, McLean JS, Lasken RS, Williamson SJ (2011). Single virus genomics: a new tool for virus discovery. PLoS ONE.

[b2] Altschul SF, Madden TL, Schaffer AA, Zhang JH, Zhang Z, Miller W, Lipman DJ (1997). Gapped BLAST and PSI-BLAST: a new generation of protein database search programs. Nucleic Acids Res.

[b3] Andrews-Pfannkoch C, Fadrosh DW, Thorpe J, Williamson SJ (2010). Hydroxyapatite-mediated separation of double-stranded DNA, single-stranded DNA, and RNA genomes from natural viral assemblages. Appl Environ Microbiol.

[b4] Angly FE, Felts B, Breitbart M, Salamon P, Edwards RA, Carlson C (2006). The marine viromes of four oceanic regions. PLoS Biol.

[b5] Bench SR, Hanson TE, Williamson KE, Ghosh D, Radosovich M, Wang K, Wommack KE (2007). Metagenomic characterization of Chesapeake bay virioplankton. Appl Environ Microbiol.

[b6] Biers EJ, Wang K, Pennington C, Belas R, Chen F, Moran MA (2008). Occurrence and expression of gene transfer agent genes in marine bacterioplankton. Appl Environ Microbiol.

[b7] Bragg JG, Chisholm SW (2008). Modelling the fitness consequences of a cyanophage-encoded photosynthesis gene. PLoS ONE.

[b11] Breitbart M, Salamon P, Andresen B, Mahaffy JM, Segall AM, Mead D (2002). Genomic analysis of uncultured marine viral communities. Proc Natl Acad Sci USA.

[b9] Breitbart M, Hewson I, Felts B, Mahaffy JM, Nulton J, Salamon P, Rohwer F (2003). Metagenomic analyses of an uncultured viral community from human feces. J Bacteriol.

[b10] Breitbart M, Felts B, Kelley S, Mahaffy JM, Nulton J, Salamon P, Rohwer F (2004). Diversity and population structure of a near-shore marine-sediment viral community. Proc R Soc Lond B Biol Sci.

[b8] Breitbart M, Thompson LR, Suttle CS, Sullivan MB (2007). Exploring the vast diversity of marine viruses. Oceanography.

[b12] Cantalupo PG, Calgua B, Zhao G, Hundesa A, Wier AD, Katz JP (2011). Raw sewage harbors diverse viral populations. mBio.

[b13] Carrigg C, Rice O, Kavanagh S, Collins G, O'Flaherty V (2007). DNA extraction method affects microbial community profiles from soils and sediment. Appl Microbiol Biotechnol.

[b14] Casjens S (2003). Prophages and bacterial genomics: what have we learned so far?. Mol Microbiol.

[b15] Clokie MRJ, Shan J, Bailey S, Jia Y, Krisch HM (2006). Transcription of a ‘photosynthetic’ T4-type phage during infection of a marine cyanobacterium. Environ Microbiol.

[b16] Dammeyer T, Bagby SC, Sullivan MB, Chisholm SW, Frankenberg-Dinkel N (2008). Efficient phage-mediated pigment biosynthesis in oceanic cyanobacteria. Curr Biol.

[b17] Dinsdale EA, Edwards RA, Hall D, Angly F, Breitbart M, Brulc JM (2008). Functional metagenomic profiling of nine biomes. Nature.

[b18] Duhaime M, Deng L, Poulos B, Sullivan M (2012). Towards quantitative metagenomics of wild viruses and other ultra-low concentration DNA samples: a rigorous assessment and optimization of the linker amplification method. Environ Microbiol.

[b19] Edwards RA, Rohwer F (2005). Viral metagenomics. Nat Rev Microbiol.

[b20] Erlwein O, Robinson MJ, Dustan S, Weber J, Kaye S, McClure MO (2011). DNA extraction columns contaminated with murine sequences. PLoS ONE.

[b21] Evans GE, Murdoch DR, Anderson TP, Potter HC, George PM, Chambers ST (2003). Contamination of Qiagen DNA extraction kits with legionella DNA. J Clin Microbiol.

[b61] Frias-Lopez J, Shi Y, Tyson GW, Coleman ML, Schuster SC, Chisholm SW, DeLong EF (2008). Microbial community gene expression in ocean surface waters. Proc Natl Acad Sci USA.

[b22] Fuhrman JA (1999). Marine viruses and their biogeochemical and ecological effects. Nature.

[b23] Fuhrman JA, Kirchman DL (2000). Impact of viruses on bacterial processes. Microbial Ecology of the Oceans.

[b24] Hellweger FL (2009). Carrying photosynthesis genes increases ecological fitness of cyanophage *in silico*. Environ Microbiol.

[b25] Henn M, Sullivan MB, Strange-Thomann N, Osburne MS, Berlin AM, Kelly L (2010). Analysis of high-throughput sequencing and annotation strategies for phage genomes. PLoS ONE.

[b26] Huse SM, Huber JA, Morrison HG, Sogin ML, Mark Welch D (2007). Accuracy and quality of massively parallel DNA pyrosequencing. Genome Biol.

[b27] Hyatt D, Chen GL, LoCascio PF, Land ML, Larimer FW, Hauser LJ (2010). Prodigal: prokaryotic gene recognition and translation initiation site identification. BMC Bioinformatics.

[b28] John SG, Mendez CB, Deng L, Poulos B, Kauffman AKM, Kern S (2011). A simple and efficient method for concentration of ocean viruses by chemical flocculation. Environ Microbiol Rep.

[b29] Kim KH, Bae JW (2011). Amplification methods bias metagenomic libraries of uncultured single-stranded and double-stranded DNA viruses. Appl Environ Microbiol.

[b30] Kim KH, Chang HW, Nam YD, Roh SW, Kim MS, Sung Y (2008). Amplification of uncultured single-stranded DNA viruses from rice paddy soil. Appl Environ Microbiol.

[b31] Lang AS, Beatty JT (2007). Importance of widespread gene transfer agent genes in alpha-proteobacteria. Trends Microbiol.

[b32] Lawrence JE, Steward GF, Wilhelm SW, Weinbauer MG, Suttle CA (2010). Purification of viruses by centrifugation. Manual of Aquatic Viral Ecology.

[b34] Lindell D, Sullivan MB, Johnson ZI, Tolonen AC, Rohwer F, Chisholm SW (2004). Transfer of photosynthesis genes to and from *Prochlorococcus* viruses. Proc Natl Acad Sci USA.

[b33] Lindell D, Jaffe JD, Johnson ZI, Church GM, Chisholm SW (2005). Photosynthesis genes in marine viruses yield proteins during host infection. Nature.

[b36] McHardy AC, Rigoutsos I (2007). What's in the mix: phylogenetic classification of metagenome sequence samples. Curr Opin Microbiol.

[b35] Mann NH, Cook A, Millard A, Bailey S, Clokie M (2003). Bacterial photosynthesis genes in a virus. Nature.

[b37] Morgan JL, Darling AE, Eisen JA (2010). Metagenomic sequencing of an *in vitro*-simulated microbial community. PLoS ONE.

[b38] Niu BF, Fu LM, Sun SL, Li WZ (2010). Artificial and natural duplicates in pyrosequencing reads of metagenomic data. BMC Bioinformatics.

[b39] Noble RT, Fuhrman JA (1998). Use of SYBR Green I for rapid epifluorescence counts of marine viruses and bacteria. Aquat Microb Ecol.

[b40] Rattei T, Arnold R, Tischler P, Lindner D, Stumpflen V, Mewes HW (2006). SIMAP: the similarity matrix of proteins. Nucleic Acids Res.

[b41] Rohwer F (2003). Global phage diversity. Cell.

[b43] Sharon I, Tzahor S, Williamson S, Shmoish M, Man-Aharonovich D, Rusch DB (2007). Viral photosynthetic reaction center genes and transcripts in the marine environment. ISME J.

[b42] Sharon I, Battchikova N, Aro EM, Giglione C, Meinnel T, Glaser F (2011). Comparative metagenomics of microbial traits within oceanic viral communities. ISME J.

[b44] Shen H, Rogelj S, Kieft TL (2006). Sensitive, real-time PCR detects low-levels of contamination by *Legionella pneumophila* in commercial reagents. Mol Cell Probes.

[b45] Stanton TB (2007). Prophage-like gene transfer agents-novel mechanisms of gene exchange for *Methanococcus, Desulfovibrio, Brachyspira*, and *Rhodobacter* species. Anaerobe.

[b46] Sullivan MB, Coleman M, Weigele P, Rohwer F, Chisholm SW (2005). Three *Prochlorococcus* cyanophage genomes: signature features and ecological interpretations. PLoS Biol.

[b47] Sullivan MB, Lindell D, Lee JA, Thompson LR, Bielawski JP, Chisholm SW (2006). Prevalence and evolution of core photosystem II genes in marine cyanobacterial viruses and their hosts. PLoS Biol.

[b48] Suttle CA (2005). Viruses in the sea. Nature.

[b49] Suttle CA (2007). Marine viruses – major players in the global ecosystem. Nat Rev Microbiol.

[b50] Temperton B, Field D, Oliver A, Tiwari B, Mühling M, Joint I, Gilbert JA (2009). Bias in assessments of marine microbial biodiversity in fosmid libraries as evaluated by pyrosequencing. ISME J.

[b51] Thurber RV, Haynes M, Breitbart M, Wegley L, Rohwer F (2009). Laboratory procedures to generate viral metagenomes. Nat Protoc.

[b53] Weinbauer MG (2004). Ecology of prokaryotic viruses. FEMS Microbiol Rev.

[b54] Williamson SJ, Rusch DB, Yooseph S, Halpern AL, Heidelberg KB, Glass JI (2008). The Sorcerer II Global Ocean Sampling Expedition: metagenomic characterization of viruses within aquatic microbial samples. PLoS ONE.

[b55] Wommack KE, Colwell RR (2000). Virioplankton: viruses in aquatic ecosystems. Microbiol Mol Biol Rev.

[b56] Wommack KE, Sime-Ngando T, Winget DM, Jamindar S, Helton RR, Wilhelm SW, Weinbauer MG, Suttle CA (2010). Filtration-based methods for the collection of viral concentrates from large water samples. Manual of Aquatic Viral Ecology.

[b57] Yilmaz S, Allgaier M, Hugenholtz P (2010). Multiple displacement amplification compromises quantitative analysis of metagenomes. Nat Methods.

[b58] Yooseph S, Sutton G, Rusch DB, Halpern AL, Williamson SJ, Remington K (2007). The Sorcerer II Global Ocean Sampling expedition: expanding the universe of protein families. PLoS Biol.

[b59] Yoshida T, Claverie J, Ogata H (2011). Mimivirus reveals Mre11/Rad50 fusion proteins with a sporadic distribution in eukaryotes, bacteria, viruses and plasmids. Virol J.

[b52] van der Zee A, Peeters M, de Jong C, Verbakel H, Crielaard JW, Claas EC, Templeton KE (2002). Qiagen DNA extraction kits for sample preparation for legionella PCR are not suitable for diagnostic purposes. J Clin Microbiol.

[b60] Zerbino DR, Birney E (2008). Velvet: algorithms for de novo short read assembly using de Bruijn graphs. Genome Res.

