# Supplementary material for: Evaluation of methods to concentrate and purify ocean virus communities through comparative, replicated metagenomics
Source: Environ Microbiol. 2013 May;15(5):1428–40. doi: 10.1111/j.1462-2920.2012.02836.x (PMC3655615; doi:10.1111/j.1462-2920.2012.02836.x)
Supplement: Supplementary file 3 [file emi0015-1428-SD3.doc]

**Supplemental Tables**

Supp. Table 1. The results of a one-way analysis of variance for the top taxonomic levels based on rank abundance in the samples by superkingdom, family, and genus. Significant results are shown in bold. Abbreviations are used for each method: FC= FeCl_3_ CsCl+DNase, FS= FeCl_3_ Sucrose+DNase, FD= FeCl_3_ DNase, TC= TFF and CsCl+DNase, and TF= TFF CsCl+DNase.

| **Taxonomy Level** | **Taxonomy Name** | **Type III Sum of Squares** | **df** | **Mean Square** | **F** | **Sig.** | **Difference in Means** |
| --- | --- | --- | --- | --- | --- | --- | --- |
| Superkingdom | Archaea | 1.62E+02 | 3 | 5.39E+01 | 1.1111 | 0.400 |  |
| Superkingdom | Bacteria | 1.36E+06 | 3 | 4.55E+05 | 0.8908 | 0.486 |  |
| Superkingdom | Eukaryota | 8.81E+04 | 3 | 2.94E+04 | 1.0647 | 0.417 |  |
| Superkingdom | Viruses | 2.19E+07 | 3 | 7.29E+06 | 3.001 | 0.095 |  |
| family | Myoviridae | 2.08E+07 | 3 | 6.94E+06 | 1.238 | 0.358 |  |
| family | Podoviridae | 9.50E+06 | 3 | 3.17E+06 | 4.376 | **0.042** | TC < FC |
| family | Siphoviridae | 5.53E+05 | 3 | 1.84E+05 | 0.623 | 0.620 |  |
| family | Rhodobacteraceae | 8.01E+05 | 3 | 2.67E+05 | 0.542 | 0.667 |  |
| family | Phycodnaviridae | 1.24E+06 | 3 | 4.12E+05 | 20.284 | **0.000** | TC < FD, FC, FS |
| family | Daphniidae | 5.84E+03 | 3 | 1.95E+03 | 0.146 | 0.930 |  |
| family | Burkholderiaceae | 4.22E+04 | 3 | 1.41E+04 | 1.497 | 0.288 |  |
| family | Bradyrhizobiaceae | 1.25E+05 | 3 | 4.18E+04 | 2.864 | 0.104 |  |
| family | Desulfovibrionaceae | 2.95E+04 | 3 | 9.83E+03 | 1.923 | 0.205 |  |
| family | Bacteroidaceae | 8.13E+03 | 3 | 2.71E+03 | 0.834 | 0.512 |  |
| genus | T4-like viruses | 2.05E+07 | 3 | 6.82E+06 | 1.283 | 0.344 |  |
| genus | T7-like viruses | 1.32E+06 | 3 | 4.41E+05 | 4.234 | 0.046 |  |
| genus | Candidatus Pelagibacter | 2.16E+03 | 3 | 7.19E+02 | 0.927 | 0.471 |  |
| genus | Synechococcus | 4.83E+05 | 3 | 1.61E+05 | 10.993 | **0.003** | TC < FD and FS |
| genus | Prasinovirus | 3.48E+05 | 3 | 1.16E+05 | 17.631 | **0.001** | TC < FD and FS |
| genus | Roseobacter | 1.06E+05 | 3 | 3.53E+04 | 0.635 | 0.613 |  |
| genus | Daphnia | 5.84E+03 | 3 | 1.95E+03 | 0.146 | 0.930 |  |
| genus | Chlorovirus | 1.75E+05 | 3 | 5.82E+04 | 10.648 | **0.004** | TC << FD and FC |
| genus | Bradyrhizobium | 1.26E+05 | 3 | 4.19E+04 | 3.819 | 0.058 |  |
| genus | Ruegeria | 1.46E+04 | 3 | 4.87E+03 | 0.838 | 0.510 |  |
